# Understanding global mental health: a conceptual review

**DOI:** 10.1136/bmjgh-2020-004631

**Published:** 2021-03-23

**Authors:** Vian Rajabzadeh, Erin Burn, Sana Z. Sajun, Mimi Suzuki, Victoria Jane Bird, Stefan Priebe

**Affiliations:** Unit for Social and Community Psychiatry, Queen Mary University of London, London, UK

**Keywords:** mental health & psychiatry

## Abstract

**Background:**

Mental health disorders are viewed as a global concern requiring globally led approaches to address them. Since the publication of the 2007 *Lancet* series on global mental health (GMH), the term has become widespread. Over the last two decades, GMH has become increasingly affiliated with policy reform, academic courses, funding bodies and research. However, it is not always obvious how those working in the field of GMH are using the term, resulting in a lack of clarity. Therefore, work is needed to synthesise the current understanding(s) of GMH to help characterise its meaning.

**Aim:**

To synthesise the literature and identify the different ways GMH is understood.

**Method:**

A conceptual review, using a systematic search and a content analysis, was conducted to develop a conceptual framework of the different conceptual understandings of GMH.

**Results:**

We developed a conceptual framework of four understandings of GMH. These understandings of GMH are as follows: an area of research generating findings to establish a GMH evidence-base; implementation of research into practice; improving the mental health environment; learning from and supporting low-and-middle-income countries (LMICs).

**Conclusion:**

Our review proposes a simple framework, clarifying the key characteristics of the GMH landscape. The findings highlight the diversity of usage of the term in the literature, as well as present the wide scope that comprises the field of GMH. Referring to this framework may help those engaged with GMH to be more specific with which aspect of the field they are concerned with.

**Key questions**

**What is already known?**

- Global mental health (GMH) is a widely used term, affiliated with policy reform, academic courses, funding bodies and research.
- However, it is not always obvious how those engaged with GMH are using the term, and what they mean by it.

**What are the new findings?**

- Four conceptualisations of GMH were identified, highlighting the term’s wide usage as well as the diversity of engagements within the field.

**What do the new findings imply?**

- It is crucial for those engaging with GMH to better acknowledge where their work lies within the field’s wide scope.

## Introduction

Globalisation has reduced the boundaries between countries, meaning that people are allegedly engaging within one ‘global village’, yet there is a widening gap between those who benefit from knowledge and technological advancement, and those who do not.^1^ The *Global Burden of Disease report*^2^ revealed the magnitude of the global burden occupied by mental disorders, followed by the 2001 World Health Report,^3^ which highlighted the inequalities in the form of treatment gaps occurring in different countries. Collectively, these developments prompted discussion, among academics, policy makers and practitioners, around mental health being viewed a global priority. Yet more recently, and consolidating some of the principles from these earlier reports, the publication of the 2007 *Lancet* series calling for efforts in scaling up mental health services globally brought the term ‘global mental health’ (GMH) to the fore, led by psychiatrists and researchers from high-income countries (HICs).^4 5^

Over the last two decades, the term has been used to underpin research, academic training, funding programmes, policy and action. Many educational institutions have established postgraduate programmes dedicated to GMH.^6 7^ Funding bodies dedicate substantial amounts to research into GMH. For example, Canada’s Grand Challenges has invested $C47.6 million, supporting 95 projects implemented in 32 low-middle-income countries (LMICs).^8^ The Global Challenges Research Fund (£1.5 billion) and Newton Fund (£735 million) both support research by UK institutes in partnership with countries around the globe, including GMH projects, which receive significant amounts of this funding.^9^ The Medical Research Council issued new investments amounting up to £20 million, dedicated to addressing the global burden of mental illness, especially in LMICs.^10^ The disruption caused by SARS-CoV-2 has also led to funding bodies calling for proposals to explore the effects of the pandemic on mental health globally.^11^ Despite momentum, there is no consensus around the meaning of GMH, rather an assumption that those engaging with the term are talking about the same thing.^12 13^

Although there has been an effort to characterise GMH by systematically evaluating its ‘implicit priorities’,^14^ it is not always clear how individuals and organisations engaged in GMH are using the term, resulting in a lack of clarity. While there are different ways GMH can be thought of, such as a domain within global health or as the humanitarian application of psychosocial approaches,^15 16^ no single definition can apply to all contexts. Furthermore, the term has been constrained by the criticisms and debate around what it truly means, putting it at risk of reaching an ‘impasse’^17^ and thus losing all meaning. Consequently, a conceptual framework can help map out GMH’s landscape and potentially portray the term’s meaning beyond the polemics which it is currently characterised by. It can help to demarcate GMH’s content and identify the key parameters that characterise the term, helping to differentiate it from similar fields, as well as help guide evaluation and monitoring of GMH-related activities.^12^ This review will consult the different ways the term is used in the academic literature to synthesise and identify how GMH is understood.

## Methods

### Overall approach

A conceptual review was conducted to synthesise the different conceptualisations of the term global mental health. As per the recommendations set out by Lilford and colleagues, the review involved multidisciplinary members as part of the review team and used an iterative approach.^18^ The main output of this process is to produce a conceptual framework for the relevant stakeholders, defined by Jabereen, as a set of related concepts that provide a comprehensive understanding of a phenomenon.^18 19^ This study protocol is registered in the PROSPERO database (CRD42017072594).

### Search strategy and eligibility

This review used a systematic search, and three search strategies were employed, electronic database searching, reviewing GMH journal series and hand-searching. The electronic databases search included Scopus, PubMed, Web of Science, Grey literature report and Open Grey. All databases were searched from inception to 6 May 2020, using the term ‘global mental health’, identified from the title, abstract and keywords. Harvard Psychiatry review 2012 and the *Lancet* series, 2007, 2011, 2018 were hand-searched based on the journal’s high impact factors, as well as searching for funding calls for any GMH-specific research opportunities.

**Figure 1:**
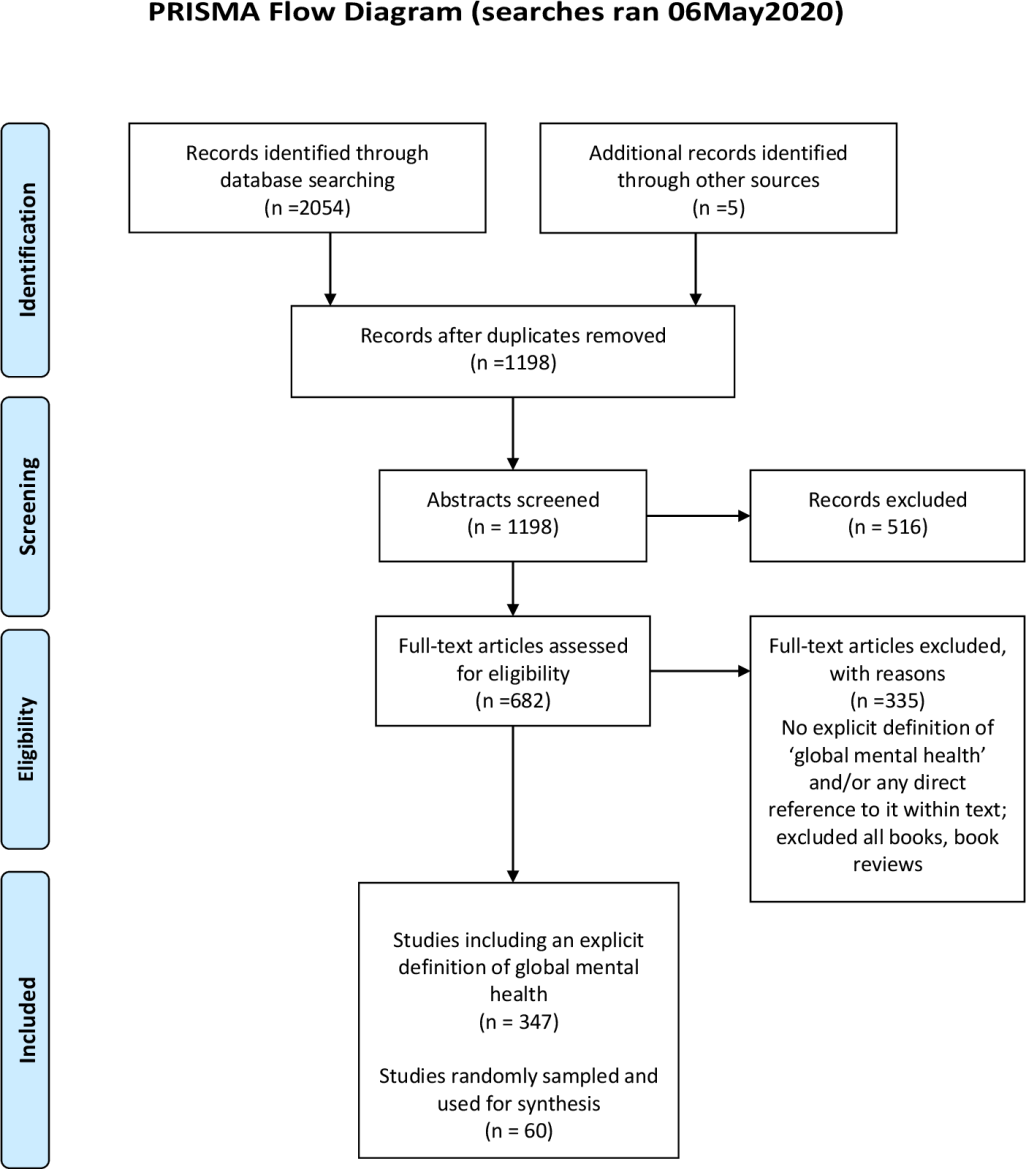
PRISMA (Preferred Reporting Items for Systematic Reviews and Meta-Analyses) flow diagram.

Eligibility was assessed on whether the authors explicitly described their understanding of GMH. VR conducted the screening process, and MS reviewed a 40% random sample. Inter-related reliability achieved an 88% concordance rate, and discrepancies were resolved among the review team. Due to the high number of papers meeting the eligibility criteria (see figure 1), a random sample of 60 articles was selected to develop the initial framework using a random number generator, which involved defining the sampling frame (1–347) and the sample size.^20^

### Data extraction and synthesis

Interpretations of GMH were extracted, as were related text, such as aims, approaches and criticisms. Content analysis was judged to be the most appropriate methodology, offering a systematic and comprehensive approach in describing a phenomenon in different contexts.^21 22^ The process followed an inductive content analysis demonstrated by Elo and Kyngäs^21^:

- Extraction of key texts was collated in a data extraction form.
- Texts were systematically, openly coded, ascribing a descriptive code.
- Descriptive codes were grouped into higher-order categories.
- Categories were collapsed based on commonalities or differences.
- Data were reduced to its fundamental characteristics, known as abstraction.

Once the framework was constructed based on the sample of 60 papers, vote counting was used to assess the validity of the framework by applying it against the remaining 287 papers (figure 1).

The multidisciplinary team included the lead researcher (VR, doctoral researcher) and five members forming an internationally diverse, mixed career stage research group (EB, SS, MS, VB, SP), including British, German, Japanese and Pakistani nationalities. The team composed of a social science doctoral student, a global health doctoral student, a global public health researcher, a psychologist, a mental health services researcher and a clinical-academic psychiatrist. EB, SS, VB and SP are involved in the coordination of a number of global health projects focusing on the delivery of co-developed community-based psychosocial interventions in LMICs—spanning across four continents, with both VB and SP acting as principal investigators. All authors are based at a WHO Collaborating Centre.

### Patient and public involvement

It was not appropriate to involve patients or the public in the design, or conduct, or reporting or dissemination plans of our research.

## Results

Based on the search strategy, 1198 unique records were retrieved. Of the identified records, 516 were excluded based on abstracts, and the remaining 682 were assessed for eligibility. After the full-text screening of the papers, 347 were identified to exhibit an explicit definition of GMH. Sixty of these were randomly selected and used to synthesise the conceptualisations of GMH. The 60 papers comprised research articles (n=18); comments, editorials or correspondence (n=16); GMH series articles (n=6); reviews (n=6); original articles (n=6); debates (n=2); case study or report (n=2); a symposium article (n=1); a thematic paper (n=1); study protocol (n=1); introduction (n=1). All 60 papers were published between 2007 and 2020, the majority were from the UK (n=28), the USA (n=14) and Europe (n=6), with the remaining papers comprising research from South Africa (n=4), Canada (n=3), Australia (n=1), India (n=1), Norway (n=1), Panama (n=1) and Switzerland (n=1).

### A conceptual framework for global mental health

**Figure 2:**
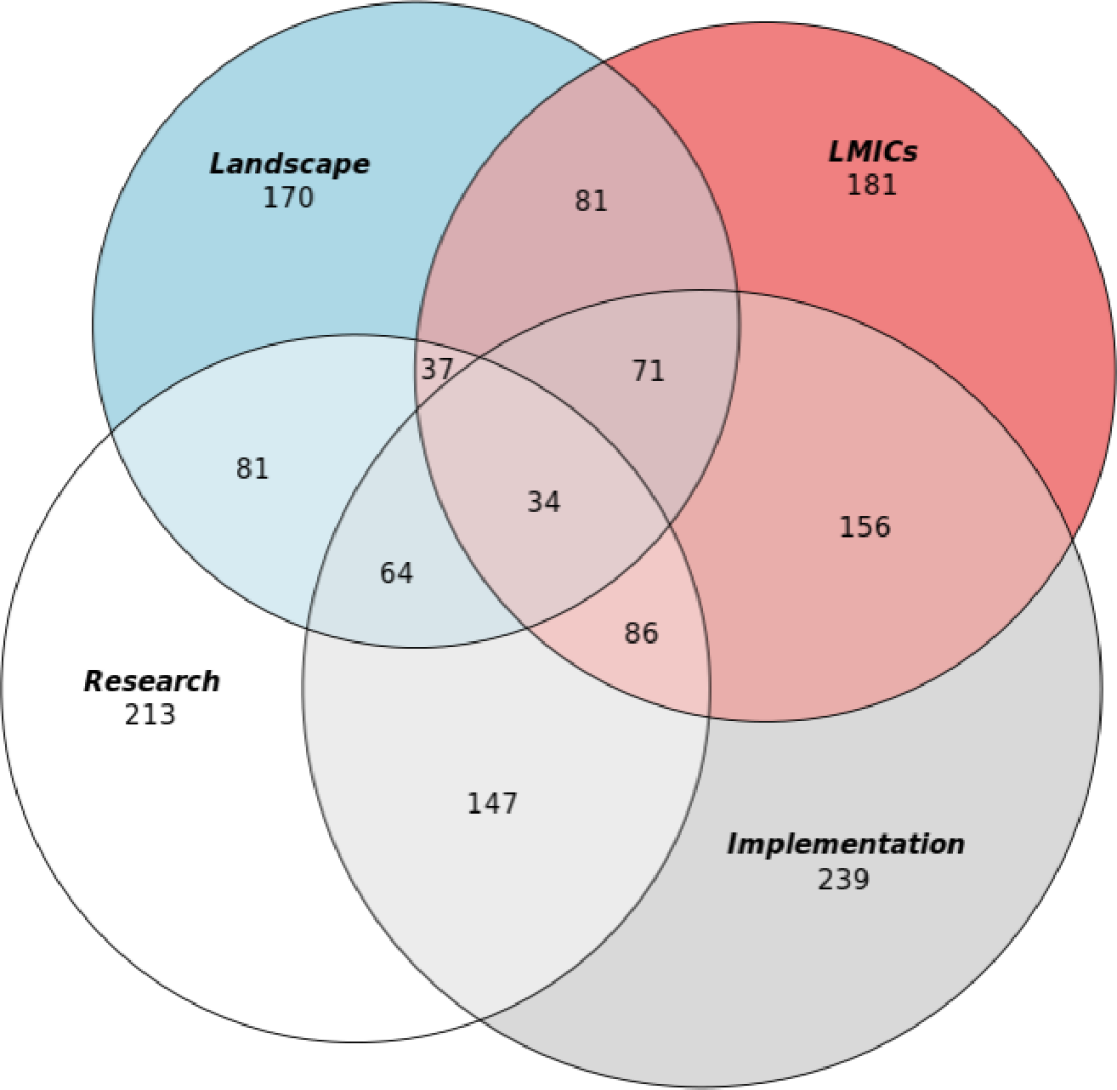
Venn diagram displaying all 347 papers and identified conceptualisations. LMICs, low-and-middle-income countries.

Four conceptualisations of GMH were derived from the 60 randomly selected papers (online supplemental appendix 1). The validity of the framework was assessed with the remaining included papers, by using vote counting, indicating how papers exhibit more than one understanding of the term (table 1). Vote counting concluded that all 347 articles used more than one conceptualisation of GMH, as research (n=213), as implementation (n=239), as landscape (n=170) and LMICs (n=181). Figure 2 displays the results from the vote counting through a Venn diagram. Table 2 portrays each conceptualisation used by the 60 papers.

10.1136/bmjgh-2020-004631.supp1 Supplementary data

**Table 1:** Understandings of global mental health

Themes	Number (%) of 347 studies identifying the themes
Globalising mental health research	213 (61.4)
Global mental health is the implementation	239 (68.9)
Improving the mental health landscape	170 (50.0)
Learning from and supporting LMICs	181 (52.2)

LMICs, low-and-middle-income countries.

**Table 2:** Included papers and identified conceptualisations

	Conceptualisations of global mental health			
	Globalised mental health research	Global mental health is implementation	Improving the mental health landscape	Learning from and supporting LMICs
Lancet Global Mental Health Group^34^	**✓**	**✓**	**✓**	**✓**
Patel *et al*^33^	**✓**	**✓**	**✓**	**✓**
Patel and Sartorius^39^	**✓**	**✓**	**✓**	**✓**
Summerfield^57^	**✓**			**✓**
Patel and Thornicroft^40^	**✓**	**✓**		**✓**
Patel and Prince^15^	**✓**	**✓**	**✓**	**✓**
Cutcliffe^23^	**✓**			
Murray *et al*^44^	**✓**	**✓**		**✓**
Baumgartner *et al*^25^	**✓**	**✓**	**✓**	**✓**
Petersen *et al*^73^		**✓**	**✓**	**✓**
Skovdal^59^	**✓**	**✓**		**✓**
Swartz^50^	**✓**			**✓**
Braathen *et al*^51^	**✓**	**✓**	**✓**	**✓**
Wildeman^76^			**✓**	
Bartlett *et al*^52^	**✓**	**✓**	**✓**	
Baumgartner and Burns^72^		**✓**	**✓**	**✓**
Barkil-Oteo *et al*^83^			**✓**	
Das^68^		**✓**	**✓**	**✓**
Ecks and Basu^55^	**✓**		**✓**	**✓**
Susser and Patel^26^	**✓**	**✓**	**✓**	**✓**
Jacob and Patel^27^	**✓**	**✓**		**✓**
Eaton *et al*^78^			**✓**	**✓**
Mills^60^	**✓**	**✓**		**✓**
Murray *et al*^47^	**✓**	**✓**		**✓**
Bemme and D’souza^28^	**✓**			**✓**
Asher *et al*^29^	**✓**	**✓**	**✓**	**✓**
Kohrt *et al*^20^	**✓**			
Pinto da Costa^79^			**✓**	
Jain and Orr^53^	**✓**	**✓**	**✓**	
Alarcón^30^	**✓**	**✓**	**✓**	
Bracken *et al*^58^	**✓**			
Datta^82^			**✓**	
Kidd *et al*^45^	**✓**	**✓**		**✓**
Magidson *et al*^81^			**✓**	
Orešković^24^	**✓**	**✓**		**✓**
Swerdfager^56^	**✓**		**✓**	
Tennyson *et al*^31^	**✓**	**✓**		**✓**
Varma^67^		**✓**		**✓**
Weinmann and Koesters^69^		**✓**		**✓**
Barbui *et al*^35^	**✓**			**✓**
Gire *et al*^63^	**✓**	**✓**		
Grigaite^66^		**✓**		**✓**
Howell *et al*^80^			**✓**	
Murphy *et al*^75^		**✓**	**✓**	
Mejia *et al*^62^	**✓**			
Taylor^54^	**✓**	**✓**	**✓**	**✓**
Asher *et al*^36^	**✓**	**✓**		**✓**
Carr^77^			**✓**	
Frankish *et al*^74^		**✓**	**✓**	**✓**
Hanlon *et al*^37^	**✓**		**✓**	**✓**
Tiley and Kyriakopoulos^38^	**✓**	**✓**	**✓**	**✓**
Priebe *et al*^64^	**✓**	**✓**	**✓**	**✓**
Hall *et al*^46^	**✓**			**✓**
Iemmi^43^	**✓**			**✓**
Kong and Singh^48^	**✓**	**✓**		**✓**
Kumar^42^	**✓**	**✓**	**✓**	**✓**
Lovell and Diagne^32^	**✓**	**✓**		
Raghavan *et al*^41^	**✓**		**✓**	
Burgess *et al*^71^		**✓**	**✓**	**✓**
White^61^	**✓**			

LMICs, low-and-middle-income countries.

### Globalising mental health research

Many authors present GMH as a specific field of research that aims to generate findings to develop and expand an effective evidence-base for global practice and guide policy towards making more informed decisions at the local, national and international levels.^15^

Patel and Prince, alongside other researchers, have acknowledged that a global response is needed to address mental health issues that have arisen due to the effects of globalisation.^15 23–32^ Supporters of the GMH movement have not only highlighted the ubiquitous nature of mental health issues; they have also illuminated the global disparity in access to treatment being notably wide in LMICs.^15 33–38^ In response to these wide treatment gaps, the GMH movement has identified scaling up treatment and delivery as an urgent research priority, particularly pressing in LMICs.^23 39 40^ Raghavan *et al*^41^ emphasised the importance of addressing the mental health of migrant communities, acknowledging that exploring ways to approach culturally diverse communities living in developed countries due to migration as a part of the GMH’s research agenda. This view is supported by other researchers that seek to address mental health issues that exist beyond the boundaries of LMICs and move towards a more inclusive GMH field.^15^

Orešković emphasises the role of global research collaboration to incorporate new ways of thinking,^24^ mostly demonstrated by partnerships between LMIC and HIC institutions.^42–45^ ^37 39 42 43^ Collaboration offers one approach to strengthening the research capacity of LMICs and subsequently improve their contribution to the GMH evidence-base.^31 35 37–39 44^ Critical evaluation of how GMH research is conducted has been emphasised by many researchers.^26 39^ More specifically, the assessment of global partnerships has shed light on the crucial role that non-specific factors, such as models of leadership, collaboration and contextual factors, play in forming effective global research partnerships.^15 42 43 45 46^ These findings can help refine future international collaborations and improve implementation, primarily when research is conducted in widely different cultural settings.^15 38 39 44 45 47^ More recently, the notion of mutual learning has been promoted widely, contending it as a crucial aspect of these collaborations, moving away from the one-directional process that has characterised past partnerships.^15 26 42 44 48 49^

From the perspective of academics aiming to develop GMH as a research field, the purpose is to create a global community, generating and translating findings from a diverse range of cultural settings, moving away from a traditional ‘silos’ approach. Consequently, there is a desire to see the cross-cultural adaptation of classifications and assessments of mental disorders, which are needed to facilitate research in culturally different contexts and allow for a global comparison.^50–57^

GMH researchers demonstrate a more integrative, resourceful and pluralistic approach to solving mental health issues shared worldwide by seeking novel ideas and solutions to address them.^15 24 46^ GMH is a highly interdisciplinary research area, benefiting from evidence generated from disciplines including epidemiology, geography and anthropology.^15 20 26 27 29–33 40^ The field of GMH is using more anthropological methodologies, such as ethnographies and participatory approaches, to capture more nuanced data of the experience of mental health disorders.^25 50–58^ Jain and Orr discussed how the use of ethnography in GMH can help characterise different mental health perspectives in a diverse range of settings.^53^

Summerfield, along with other anthropological or cross-cultural psychiatrists, has accused the GMH agenda of relying on Western psychiatry and ignoring the role of culture, context and experience in individuals with mental health disorders.^15 20 50 52 53 57–60^ This criticism emphasised the need for more culturally resonating research by incorporating local relevant knowledge and conceptualisations of mental health, as well as the narratives of key stakeholders impacted by mental disorders.^15 23 27 28 32 40 51–53 56–59 61 62^ In response to the criticism around the absence of local voices, GMH researchers have developed innovative, cost-effective interventions that prioritise local stakeholders.^33 63–65^ Asher *et al* demonstrated how collaborating with alternative healing forms, such as spiritual or religious, can be incorporated into the GMH treatment framework.^36^

GMH demonstrates an extending scope for research by shifting its focus to factors that maintain and sustain mental health, as well as looking at determinants.^24 51 61^ Research that is being conducted indicates a shift towards an epistemological pluralism, where no one dominant paradigm is favoured over another, to accommodate more diverse perspectives, which can help achieve a GMH evidence-base comprising clinical, social and cultural frameworks.^24 30 52 54^

### Global mental health is implementation

Other supporters of the term GMH, particularly those involved in implementing healthcare programmes, use the term to imply the activities undertaken to promote the development of mental health infrastructure, especially in LMICs.^27 30 39 52 53^ GMH advocate’s key focus is to address the lack of mental health infrastructure, particularly in LMICs, by building mental health systems’ capacity.^26 39^

The sentiment of action has been endorsed through ‘scaling-up’ mental health services, which is defined as increasing the provision of evidence-based services for individuals with mental disorders, particularly in LMICs.^15 27 30 34 39 45^ Scaling up interventions has been demonstrated in two distinct ways: integrating programmes into existing health systems and replacing institutional care^66–68^ ⁠with continued community care.^15 29 69^

It has become more recently apparent that research exploring the applicability, feasibility and sustainability of implementing interventions is limited.^31 38 44 47 69^ LMICs, in particular, experience many barriers preventing the integration of interventions into existing health systems, including limited government support, scarce mental health professionals, inadequate research capacity and poorly developed mental health systems.^15 33 38 47 69^ Therefore, the implementation aspect of GMH aims to understand more about how mental health interventions can be sustainability integrated into different settings, particularly in LMICs.^44 45 47^

Incorporating the evaluation of implementation programmes can help identify the barriers and facilitators to improve the uptake of interventions into health systems further down the line.^31 38 47 69^ Moreover, ensuring that these programmes are as much about strengthening research capacity as they are about the effectiveness and efficacy of the intervention is crucial.^36 39 47 64^ LMICs receive minimal governmental support, but they do have access to funding through research to improve mental health infrastructure, usually governed by HIC institutes.^69^ Yet, there are many barriers preventing the effectiveness of externally led programmes in actually improving LMIC mental health infrastructure,^38 42 69 70^ further supporting the need for locally driven programmes in addressing locally rooted issues with locally led solutions.^44 53^

Burgess and colleagues promote the role of communities in delivering interventions, allowing them to advance and address some of the socio-structural determinants of mental illness, and become a key asset of GMH.^71^ Improving community care provision is one way of scaling up mental health practices and has been targeted at LMICs.^25 26 29 53 59 71–74^ Community participation has been described as a strategy to improve mental health services’ cultural competency by increasing the mental health literacy of community health workers.^71 73^ Having closer links to the community can improve mental health awareness and help identify the sociocultural determinants of mental health disorders and protective factors.^25 26 30 59 71–74^ Task-shifting, where lay health workers are trained to deliver interventions, has been employed to solve community health professionals’ scarcity.^25 26 40 68 69 73 74^ Other practices promoted by GMH practitioners include social inclusion programmes^25 26 71 72^ and integrating mental healthcare into primary care.^24 29 34 68^

GMH practice has demonstrated expanding scope in collaboration between traditional forms of treatment, such as spiritual healing, and the professional sector.^36 51^ Besides collaborating with alternative forms of therapy, GMH seeks to harness innovative technologies to support treatment, diagnosis and education.^63 64 69 74^⁠ For example, Murphy and colleagues demonstrate how a peer-to-peer e-learning intervention can improve learning in low-resource areas and facilitate a cross-cultural awareness of mental health.^75^

Within the context of those promoting the growth of mental health infrastructure globally, GMH is characterised by a global–local debate, where there is the generalisable evidence-based biomedical approach to services versus the more empathetic locally embedded service approach.^30 32 45 48 53 60^ The global approach has been described as the propagation of Western psychiatry, which is at risk of stifling cultural alternatives to mental healthcare.^30 52 54 60^ In contrast, the local approach calls for a bottom-up approach to mental healthcare and prioritises local knowledge and stakeholders engaged in designing and delivering care.^30 32 69^

### Improving the mental health landscape

GMH policy recognises a changing world through interconnectedness and shared mental health concerns. Therefore, in response to these contemporary issues, policy makers affiliated with GMH seek to develop the appropriate mental health-enhancing policies that can facilitate supportive environments, strengthen community participation in mental health and reorient mental health services.^51 71 76^ Also, GMH advocates recognise that a shared global response is required to develop adequate mental health infrastructure and support research addressing mental health prevention and promotion.

GMH supporters have described the term as a social movement advocating for global change in how mental health is understood and how mental health disorders are treated.^15 26 33 74^ As part of changing the way we view mental health, Eaton *et al* and others advocate for policies that recognise social inclusion, protect human rights of vulnerable individuals and reduce the discrimination of those living with mental disorders.^26 39 53 56 76–80^ GMH aims to develop policies that recognise this inclusivity, by prioritising vulnerable groups and incorporating the perspectives of service users and other relevant stakeholders.^52 76–78^

The actors coordinating the movement have raised the profile of mental health against the global backdrop by framing it as a global health issue to help it gain attention and resources against other globally prevalent diseases.^26 78 80^ In terms of mental health policies, GMH researchers and practitioners are concerned with identifying where there are opportunities and barriers for policy reform in ways that can improve mental healthcare treatment and prevention, especially in LMICs.^33 34 38 39 42 68 76 78^

Training towards effective leadership and management of mental health system development and expertly skilled mental health professionals with close links to the community has been promoted by GMH.^42 81^ Community participation has been endorsed in the call for scaling up service delivery.^15 25 26 51 73^ ⁠⁠The approach aims to mobilise health resources and build capacity by improving mental health literacy, providing culturally competent care and delivering psychosocial care.^15 25 29 72 73^ Extending capacity building to policy makers to aid with mental healthcare systems reform has been explored.^37^ Training and education programmes are developing culturally competent curriculums, encompassing approaches to care for marginalised populations, such as asylum seekers and migrant communities.^30 41 75 81–83^ GMH has noted a shift in focus in mental healthcare, from targeting the determinants of mental disorders through treatment to reorienting care towards more promotion and prevention.^51 64^ Priebe *et al*^64^ demonstrate how resource-oriented interventions can tap into ‘existing resources and social structure in LMICs’ as a way to promote mental health within communities.

Policy reform advocated by GMH has been subject to criticism, such that there is a divide between the universal policy promoting evidence-based approaches and policy supporting initiatives which are embedded in the context.^53 76^ Therefore, similarly, with research and implementation, there is a drive towards policy to helping more diverse, culturally relevant GMH research and practice.^53–55 80^

### Learning from and supporting low-and-middle-income countries

GMH researchers, practitioners and policy makers are guided by where the treatment gaps are the widest, which occur primarily in LMICs.^27 39 44^ GMH is concerned with targeting efforts predominantly at LMICs, as a way to support global development.^33 34 74 78^ This understanding of GMH considers the previous conceptual understandings and articulates a sense of priority towards LMICs and less-resourced areas in general.

One of the goals of GMH is to develop a globally representative evidence-base, meaning that all countries can contribute their findings to the GMH evidence-base.^57^ Yet, it is well recognised that LMICs experience wide research gaps, where there is limited original research output from these countries to contribute to the GMH evidence-base effectively⁠.^37–39^ In addition to constrained research capacity, the access and use of evidence supporting mental health practice are an ongoing challenge for LMICs.^29 35 37 38^ Therefore, a pertinent aim for GMH is to strengthen research capacity in LMICs to close the research gaps and support those countries achieving autonomy over setting their research agendas.^34 37–39 44 45 69^

Although Frankish and colleagues claim that the GMH movement is to serve all people worldwide,^74^ most of the evidence suggests that the focus is, in actuality, on LMICs.^15 26 33 40^ Consequently, anthropologists have criticised the movement as reprising the dynamics of the colonial era by exporting Western concepts and interventions to culturally different contexts and the unidirectional knowledge flow occurring in global partnerships.^15 28 50 59 60^ Yet, in response to this criticism, there has been an increasing emphasis on the process of mutual learning, especially between HIC and LMIC academic institutions, where both sides of the partnership cultivate an understanding.^15 24 26 46 48^ Furthermore, GMH researchers are growing and expanding frameworks that underpin mental health treatment to incorporate more cost-effective, innovative and traditional therapies, which are often located in LMICs, due to the lack of formal care available.^36 51 54 57^ Research conducted in LMICs can offer opportunities for reverse innovation where creativity can flourish in the context of limited resources, therefore providing an environment for innovation.^64^

The integration of programmes into existing health systems has been mostly directed at LMICs to improve and develop their mental healthcare infrastructure.^24 36 38 44 47^ More effort is needed to overcome the challenges faced by LMICs, particularly around acceptability, feasibility and sustainability of interventions.^31 36 38 44 47^ Strengthening community care^25 68 69 71 73 84^ offers an alternative approach to institutionalised care and improves service provision, as well as adding variety to the care available in LMICs.^66–69^ Task-sharing is a solution directed at LMICs to address the issues of limited human resources due to the effects of globalisation.^34 40 69^

Governments do not adequately prioritise mental health in LMICs, as well as being highly stigmatised, these countries lack the appropriate legislation to guide mental health services and programmes.^15 39 43 55 69^ As previously mentioned, global research partnerships offer one way of redistributing resources to LMICs^31 38 44 69^ to improve mental health research and reduce the stigma surrounding mental disorders.^26 29 40 72 73^ These partnerships face challenges of equity and overcoming the power dynamics in these relationships, usually between LMICs and HIC academic institutions.^42^

## Discussion

### Main findings

The present study synthesised four closely related conceptualisations of GMH. First, as research, GMH is defined as a critical investigation that can generate new knowledge that can help to address mental health issues requiring a globally led response, by guiding practice and policy. The findings indicate that this conceptualisation of GMH has evolved over time, responding to criticism, through involving local stakeholders in the research process. Through a multidisciplinary approach, researchers can integrate their expertise to help solve problems. Second, implementation in GMH has also evolved through shifting its focus from institutional forms of treatment to more community-based care, and at the same time providing care that is more locally relevant and working from the bottom upwards. Third, improving the mental health landscape describes the engagement of policy working in GMH to create an environment that prioritises and protects individuals with mental disorders, globally. Lastly, it is evident that the priority, of actors engaged with GMH, is to support LMICs, while being wary of repeating the conditions of colonialism and viewing global research partnerships as an opportunity for creativity and innovation. Almost all actors engage with more than one conceptualisation of the term shown in figure 2.

### Strengths and limitations

This review has several strengths and limitations. This is the first review conceptualising how GMH is understood. Given that there are four understandings of GMH taken from the literature, these findings support the discussion around characterising the field beyond the debates that currently surround it.^17^ The methodology accommodated an iterative process, allowing the review team to trace back to the source text supporting the concepts, when it was necessary for further discussion or clarification. Content analysis offers a flexible, pragmatic approach, in distilling a large number of articles into their fundamental characteristics, in this case, four clear concepts.^22 85^ Comparing the four conceptualisations with the remaining papers, as outlined in the Methods section, through vote counting, reinforced the validity of the framework. However, this review has several limitations. Despite conducting a comprehensive search of the literature, the literature is sourced predominantly from research; therefore skewing the findings towards a more research focus. Although the benefits of using a multidisciplinary team added to the rigour, the concepts derived offer one interpretation of the literature reviewed, and perhaps alternative conceptualisations could have arisen depending on how the ‘components’ were articulated.^86^ Restricting to the use of English language papers only may have limited the search, and therefore reduced the possibility for different cultural perspectives in the development of the concepts. A further limitation is that there is an under-representation of LMIC authors contributing to research publications that originate from LMICs^87^; therefore, despite conducting a systematic search, there will be a bias towards a more Western perspective on how GMH is understood.

### Interpretation and comparisons with existing literature

Despite being connected to numerous activities in research, practice and policy,^88–90^ there is equally no shared understanding of the term global health, nor does it have the appropriate frameworks to support such activities.^88 91 92^ Yet, many definitions of global health do exist.^92–94^ Comparatively, with the findings in this review, global health exhibits multiple roles, each one serving a different purpose and involving different actors.^94^ As a discipline, global health seeks for global cooperation in finding solutions for health issues worldwide.^92^ The notion of forming a global community resonates with the understanding of GMH in that it aims to translate and generate findings from a range of settings to create a culturally relevant evidence-base. Global health acknowledges the transcendental nature of health determinants in the same way GMH does,^92^ as well as the potential for discovering novel therapies that can be adapted and implemented in different settings. Furthermore, global health’s primary focus is to achieve equity in health for all worldwide^92 95^ and similarly with GMH, it is accused of doing this using predominantly Western approaches to treatment.^96 97^

The meaning of global health will vary depending on the view of the researchers or practitioners working within it,^90^ which is apparent considering the different understandings of GMH. Although debates within the field have helped drive it,^17^ these findings may offer a novel way of viewing GMH that exists beyond the local–global divide, which could foster ideas and perspectives that emerge along its continuum. Furthermore, the findings support the argument for greater attention of the local–global relationship, particularly in the context of the role that local communities play in driving some of the core aims of GMH.^98^

The understanding of global health has shifted over time, evolving its agenda, from a biomedical focus towards encapsulating a broader interdisciplinary approach, such as linking with anthropology to help form a more holistic view of health on a global scale.^90^ This changing agenda is notable in the current findings, where GMH research seeks to accommodate novel and innovative ways to address mental health issues and inequities, and work towards a more nuanced landscape.^99^

### Implications for research and practice

Although the review did not attempt to create a new definition for GMH, it has provided a simple framework, which offers a detailed background of what is currently being associated with the term. First, the different conceptualisations presented in this review may remind actors engaging with GMH of its wide usage within the realm of academia, and may present authors with a useful classification scheme to refer to. In addition to the term’s varied usage, the framework demonstrates the diversity that exists within the field, such as through its capacity to adopt epistemological pluralism, as well as the potential for the field to become integrative in the manner it addresses mental health problems globally. For example, the potential to develop existing frameworks of formal care to accommodate alternative forms of healing, which are more prevalent in LMICs.^54 97 98^ Alongside epistemological diversity, the framework emphasises the interdisciplinary nature of GMH and the capacity for potential linkage with other disciplines such as anthropology and geography.^52 54^⁠ It is necessary that those working in the GMH field better acknowledge where their efforts specifically contribute along the continuum of engagement, therefore referencing the proposed framework may help encourage this.

## Conclusion

This conceptual review has synthesised and identified four overlapping ways GMH is understood in the literature. The simple framework outlines the key characteristics of the GMH landscape, which may serve as a useful guide for monitoring and evaluation. The findings emphasise not only the broad usage of the term within academic literature but also the diversity existing within the field of GMH, which is not confined to the limits of the local–global debate. Referencing a framework like this may help those engaging with the field to clearly delineate where their work fits within the scope of GMH.
