# Supplementary material for: Understanding global mental health: a conceptual review
Source: BMJ Glob Health. 2021 Mar 23;6(3):e004631. doi: 10.1136/bmjgh-2020-004631 (PMC7993328; doi:10.1136/bmjgh-2020-004631)
Supplement: Supplementary data [file bmjgh-2020-004631supp001.pdf]

Appendix

Appendix 1. Characteristics of included papers

ID	Author	Title	Journal	Type of paper	Country[1]
1	Chisholm et al. (2007)	Global mental health 6 - Scale up services for mental disorders: a call for action	The Lancet	Series article	UK
2	Patel et al. (2007)	Treatment and prevention of mental disorders in low-income and middle-income countries	The Lancet	Series article	UK
3	Patel & Sartorius (2008)	From science to action: the Lancet series on global mental health	Current Opinion in Psychiatry	Commentary	UK
4	Summerfield (2008)	How scientifically valid is the knowledge base of global mental health?	British Medical Journal	Research article	UK
5	Patel & Thornicroft (2009)	Packages of care for mental, neurological, and substance use disorders in low- and middle-income countries: PLoS Medicine Series	PLoS Medicine	Series article	UK
6	Patel & Prince (2010)	Global mental health: a new global health field comes of age.	JAMA	Commentary	UK
7	Cutcliffe (2011)	Global mental health in an interconnected, reciprocal world.	Archives of psychiatric nursing	Editorial	US
8	Murray et al. (2011)	Building capacity in mental health interventions in low resource countries: an apprenticeship model for training local providers.	International journal of mental health systems	Case study	US
9	Baumgartner et al. (2012)	Measuring social integration in a pilot randomized controlled trial of critical time: intervention-task shifting in Latin America.	Cadernos saude coletiva	Research article	US
10	Petersen et al. (2012)	Understanding the benefits and challenges of community engagement in the development of community mental health services for common mental disorders: Lessons from a case study in rural South African subdistrict site	Transcultural Psychiatry	Series article	South Africa
11	Skovdal (2012)	Pathologising healthy children? A review of the literature exploring the mental health of HIV-affected children in sub-Saharan Africa	Transcultural Psychiatry	Series article	Norway

12	Swart (2012)	An unruly coming of age: The benefits of discomfort for global mental health	Transcultural Psychiatry	Series article	South Africa
13	Braathen et al. (2013)	Understanding the local context for the application of global mental health: a rural South African experience.	International Health	Original article	South Africa/ Norway
14	Wildeman (2013)	Protecting rights and building capacities: challenges to global mental health policy in light of the convention on the rights of persons with disabilities.	The Journal of law, medicine & ethics	Symposium article	Canada
15	Bartlett et al. (2014)	What's in the 'treatment gap'? Ethnographic perspectives on addiction and global mental health from China, Russia, and the United States.	Medical anthropology	Original article	US
16	Baumgartner & Burns (2014)	Measuring social inclusion-a key outcome in global mental health	International Journal of Epidemiology	Review article	South Africa
17	Barkil-Oteo et al. (2014)	Teaching global mental health at home and abroad	The Lancet Psychiatry	Commentary	US
18	Das (2014)	The context of formulation of India's Mental Health Program: implications for global mental health.	Asian Journal of Psychiatry	Review article	India
19	Ecks & Basu (2014)	"We Always Live in Fear": Antidepressant Prescriptions by Unlicensed Doctors in India	Culture, medicine and psychiatry	Original article	UK
20	Susser & Patel (2014)	Psychiatric epidemiology and global mental health: joining forces	International Journal of Epidemiology	Editorial	US
21	Jacob & Patel (2014)	Classification of mental disorders: a global mental health perspective.	The Lancet	Viewpoint	UK
22	Eaton et al. (2014)	A position statement on mental health in the post-2015 development agenda	International Journal of Mental Health Systems	Debate	UK
23	Mills (2014)	Psychotropic childhoods: Global mental health and pharmaceutical children	Children and Society	Original article	UK
24	Murray et al. (2014)	Implementing evidence-based mental health care in low-resource settings: A focus on safety planning procedures	Journal of Cognitive Psychotherapy	Research article	US
25	Bemme & D'souza (2014)	Global mental health and its discontents: An inquiry into the making of global and local scale	Transcultural Psychiatry	Research article	Canada
26	Asher et al. (2015)	Development of a Community- Based Rehabilitation Intervention for People with Schizophrenia in Ethiopia	PLoS one	Research article	Ethiopia/UK

27	Kohrt et al. (2015)	Cross-cultural gene− environment interactions in depression, post-traumatic stress disorder, and the cortisol awakening response: FKBP5 polymorphisms and childhood trauma in South Asia	International review of psychiatry	Research article	US
28	Pinto da Costa (2015)	Global Mental Health: What is Your Role in This Movement?	Acta medica portuguesa	Editorial	Portugal
29	Jain & Orr (2016)	Ethnographic perspectives on global mental health	Transcultural psychiatry	Introduction	UK
30	Alarcon (2016)	Global mental health and systems of diagnostic classification: Clinical and cultural perspectives	Acta Bioethica	Research article	Peru/US
31	Bracken et al. (2016)	Primum non nocere. The case for a critical approach to global mental health.	Epidemiology and Psychiatric Sciences	Editorial	Ireland
32	Datta (2016)	The Problem with Education in Global Mental Health	Academic psychiatry	Perspective	US
33	Kidd et al. (2016)	A Multiple Case Study of Mental Health Interventions in Middle Income Countries: Considering the Science of Delivery	PLoS One	Research article	Canada
34	Magidson et al. (2016)	Massachusetts General Hospital Global Psychiatric Clinical Research Training Program: A New Fellowship in Global Mental Health	Academic psychiatry	Educational case report	US
35	Oreskovic (2016)	Breaking down the Silo Mentality in Global Mental Health: The New Role for the Schools of Public Health.	Psychiatria Danubina	Editorial	Croatia
36	Swerdfager (2016)	Theorizing resistance: Foucault, cross-cultural psychiatry, and the user/survivor movement	Philosophy, Psychiatry and Psychology	Research article	US
37	Tennyson (2016)	Challenges and strategies for implementing mental health measurement for research in low-resource settings.	International health	Review article	US
38	Varma (2016)	Disappearing the asylum: Modernizing psychiatry and generating manpower in India	Transcultural Psychiatry	Research article	US
39	Weinmann & Koesters (2016)	Mental health service provision in low and middle-income countries: recent developments.	Current opinion in psychiatry	Review article	Switzerland
40	Barbui et al. (2017)	Cochrane for global mental health	The Lancet Psychiatry	Correspondence	Italy
41	Gire et al. (2017)	mHealth based interventions for the assessment and treatment of psychotic disorders: a systematic review.	mHealth	Review article	UK

42	Grigaite (2017)	The Deinstitutionalization of Lithuanian Mental Health Services in Light of the Evidence-based Practice and Principles of Global Mental Health	SOCIALINE TEORIJA EMPIRIJA POLITIKA IR PRAKTIKA	Research article	Portugal
43	Howell et al. (2017)	The (Mis)appropriation of HIV/AIDS advocacy strategies in Global Mental Health: towards a more nuanced approach	Globalization and health	Debate	UK
44	Murphy et al. (2017)	Problem-based, peer-to-peer global mental health e-learning between the UK and Somaliland: a pilot study	Evidence Based Mental Health	Original article	UK
45	Mejia et al. (2017)	Different Strokes for Different Folks? Contrasting Approaches to Cultural Adaptation of Parenting Interventions	Prevention Science	Research article	Panama
46	Taylor (2017)	Making space for restoration: epistemological pluralism within mental health interventions in Kinshasa, Democratic Republic of Congo	Area	Research article	UK
47	Asher et al. (2018)	Global mental health and schizophrenia.	Current opinion in psychiatry	Research article	UK
48	Carr (2018)	Implementing sustainable global mental health in a fragmenting world	The Lancet	Commentary	UK
49	Frankish et al. (2018)	Mental health for all: a global goal	The Lancet	Commentary	UK
50	Hanlon et al. (2018)	Evaluating capacity-building for mental health system strengthening in low- and middle-income countries for service users and caregivers, service planners and researchers	Epidemiology and Psychiatric Sciences	Editorial	Ethiopia/UK
51	Tiley & Kyriakopoulos (2018)	Evidence-based practice in a multicultural world: changing with the times.	BJPsych international	Thematic paper	UK
52	Priebe et al. (2019)	Resource-oriented interventions for patients with severe mental illnesses in low- and middle-income countries: trials in Bosnia-Herzegovina, Colombia and Uganda.	BMC Psychiatry	Study protocol	UK
53	Hall et al. (2019)	Intersectoral collaboration for people-centred mental health care in Timor-Leste: a mixed-methods study using qualitative and social network analysis.	International journal of mental health systems	Research article	Australia
54	Iemmi (2019)	Sustainable development for global mental health: a typology and systematic evidence mapping of external actors in low-income and middle-income countries.	BMJ global health	Research article	UK

55	Kong & Singh (2019)	The ethics of global psychiatric genomics: Multilayered challenges to integrating genomics in global mental health and disability-A position paper of the Oxford Global Initiative in Neuropsychiatric GenEthics (NeuroGenE).	Neuropsychiatric Genetics	Research article	UK
56	Kumar (2019)	Championing Equity, Empowerment, and Transformational Leadership in (Mental Health) Research Partnerships: Aligning Collaborative Work With the Global Development Agenda.	Frontiers in psychiatry	Review article	UK
57	Lovell & Diagne (2019)	Falling, Dying Sheep, and the Divine: Notes on Thick Therapeutics in Peri-Urban Senegal.	Culture, medicine and psychiatry	Original article	France
58	Raghavan et al. (2019)	Reflections on the use of mental health resilience concepts in migration and global mental health	International Journal of Mental Health	Commentary	UK
59	Burgess et al. (2020)	Social interventions: a new era for global mental health?	The Lancet Psychiatry	Commentary	UK
60	White (2020)	Mental wellbeing in the Anthropocene: Socio-ecological approaches to capability enhancement.	Transcultural psychiatry	Research article	UK

[1]Country of first author's academic affiliation
